# Phase II study of weekly paclitaxel and capecitabine in patients with metastatic or recurrent esophageal squamous cell carcinoma

**DOI:** 10.1186/1471-2407-11-385

**Published:** 2011-09-02

**Authors:** Tak Yun, Ji-Youn Han, Jin Soo Lee, Hyun Lee Choi, Hyae Young Kim, Byung-Ho Nam, Heung Tae Kim

**Affiliations:** 1 Lung Cancer Branch, National Cancer Center, Goyang, Republic of Korea; 2 Cancer Biostatistics Branch, National Cancer Center, Goyang, Republic of Korea

**Keywords:** paclitaxel, capecitabine, squamous cell carcinoma of the esophagus

## Abstract

**Background:**

This phase II study assessed the response rate and toxicity profile of weekly paclitaxel and capecitabine in patients with metastatic or recurrent squamous cell carcinoma of the esophagus (SCCE)

**Methods:**

Patients with histologically confirmed SCCE were treated with paclitaxel 80 mg/m^2 ^intravenously on days 1 and 8 plus capecitabine 900 mg/m^2 ^orally twice a day on days 1-14. Treatment cycles were repeated every 3 weeks until disease progression or unacceptable toxicity.

**Results:**

Between 2006 and 2009, 32 patients were enrolled. Twelve patients were chemotherapy-naïve. Twenty patients had received prior chemotherapy including platinum-based regimens. Patients received a median of 5 cycles of treatment (range, 1-12). The response rate was 75% (95%CI; 50.5~99.5%) in the first-line and 45% (95%CI; 26.9~73.1%) in the second-line. With a median follow-up of 20.7 months, median progression-free survival was 5.2 months (95% CI, 4.0 to 6.4) for all patients and median overall survival (OS) was 11.7 months (95% CI, 5.5 to 18.0) for all patients. The median OS was 14.3 months (95% CI, 10.6 to 18.0) for patients receiving therapy as 1^st ^line and 8.4 months (95% CI, 6.6 to 10.1) for those receiving as 2^nd^-line therapy. Grade 3/4 neutropenia was observed in 53.3% of the patients, which was the most common cause of dose reduction. G3 non-hematologic toxicity included stomatitis (9.4%), asthenia (6.3%), and hand-foot skin reaction (3.1%).

**Conclusions:**

Weekly paclitaxel and capecitabine is a highly active and well-tolerated regimen in patients with metastatic or recurrent SCCE in the first-line as well as second-line setting.

## Background

Esophageal cancer is characterized by poor prognosis, with 50% of patients presenting with metastatic disease at the time of diagnosis. In the remaining 50% of patients presenting initially with loco-regional disease, systemic metastatic disease will develop in the vast majority. The prognosis for patients diagnosed with advanced esophageal cancer is poor with a 5-year survival of 10-15% from diagnosis [1]. Conventional single agents active in esophageal cancer include cisplatin, 5-FU, etoposide, and mitomycin, with response rates ranging from 15% to 25% [1-4]. The two-drug combination of cisplatin and 5-FU has been the standard regimen for two decades, with a 25-35% response rate in metastatic disease. However, complete responses are rare, median duration of response is usually short, and the median survival time is only 6-10 months [5]. New regimens such as paclitaxel-cisplatin-5FU and irinotecan-cisplatin have shown promising anti-tumor activity in phase II trials [2,6]. Since first-line therapies are not curative, patients eventually experience disease progression. Once the disease progresses, the median survival time is very short. No regimen can be considered as standard in the second-line setting. Thus, patients with good performance status are candidates for clinical trials exploring further treatment options.

Paclitaxel is used at a dose range of 135 to 200 mg/m^2 ^over 3 hours in patients with other solid tumors such as non-small cell lung cancer. However, toxicities have been excessive when combined with cisplatin. Weekly paclitaxel showed comparable efficacy to that of 3-weekly paclitaxel, while having a lower incidence of myelosuppression and neurotoxicity [7,8].

Capecitabine is an orally administered fluoropyrimidine that is converted by 5-FU by thymidine phosphorylase (TP), preferentially in tumor tissues and has demonstrated activity as single agent in patients with gastrointestinal cancers. The tumor selectivity of capecitabine has been documented in clinical studies, where administration of capecitabine has been shown to result in approximately 2.5 times higher concentrations of 5-FU in the tumor tissue than in normal tissues [9]. Orally administered capecitabine mimics continuous-infusion of 5-FU, is well tolerated, with hand-foot syndrome and diarrhea the most common toxicities reported, and is more convenient for patients.

Taxanes upregulate the activity of TP in mouse mammary tumor cells in vitro and in xenograft models [10]. This taxane-mediated upregulation is synergistic, time dependent, and persists for up to 10 days. Thus, sequential administration of taxanes followed by capecitabine could result in enhanced efficacy of capecitabine. The different toxicity profiles of the two drugs and preclinical synergy provide the rationale for evaluating the combination of capecitabine with paclitaxel clinically. Also, taxanes plus capecitabine were reported to be highly active against non-small cell lung cancer [11], breast cancer [12], and gastric cancer [13]. Schedule optimization based on the upregulation of TP may result in a greater therapeutic index, thus allowing for the determination of the most advantageous way of combining these agents. In preclinical experiments, upregulation of TP activity was noted within 4 days of taxane treatment, and the effect was maximal at about 6-8 days [10]. Hence, a weekly schedule of paclitaxel would provide improved synergy for administration in combination with capecitabine. Considering synergistic activity and the different toxicity profiles of paclitaxel and capecitabine, we conducted a phase II trial evaluating the efficacy and safety in patients with esophageal squamous cell carcinoma.

## Methods

### 1. Study Population

Patients with metastatic or recurrent squamous cell carcinoma of the esophagus that had been histologically confirmed were eligible. Additional inclusion criteria were as follows; 1) at least 18 years old, 2) ECOG performance status of 0 to 2, 3) measurable lesions defined as RECIST 1.0, 4) adequate bone marrow, hepatic, and renal functions, defined as WBC ≥ 3,500/mm^3^, absolute neutrophil count (ANC) ≥ 1,500/mm^3^, platelets ≥ 100,000/mm^3^, ALT or AST < 2.5 times the upper normal limit, bilirubin ≤ 1.5 times the upper normal limit, and serum creatinine ≤ 1.5 mg/dL, 5) no prior radiotherapy to measurable lesions. Patients were excluded if there was severe co-morbidity such as myocardial infarction within preceding 6 months or symptomatic heart disease including unstable angina, congestive heart failure, or uncontrolled arrhythmia, and serious concomitant infection. Patients unable to swallow the capecitabine tablets were also excluded, even after placement of a stent.

Written informed consent approved by the Institutional Review Board of National Cancer Center was obtained from all patients prior to entering the study. The study followed the Declaration of Helsinki and good clinical practice guidelines. This study is registered with http://ClinicalTrials.gov (identifier; NCT00453323).

### 2. Treatment

Paclitaxel (Padexol^®^) was provided by Shinpoong pharmaceutical company and capecitabine (Xeloda^®^) was provided by Roche. Treatment was given in the outpatient setting. It consisted of paclitaxel 80 mg/m^2 ^intravenously on days 1 and 8 and capecitabine 900 mg/m^2 ^orally twice a day on days 1-14 followed by a 1 week rest period. The Treatment cycle was repeated every 3 weeks, until disease progression or unacceptable toxicity. Patients were premedicated with dexamethasone 20 mg, pheniramine maleate 45.5 mg, and famotidine 20 mg, 30 minutes before administration of paclitaxel. For practical reasons, capecitabine doses were rounded to the nearest dose that could be administered with a combination of 500-mg and 150-mg tablets of drug. Capecitabine was given approximately 12 hours apart and taken orally with water within 30 minutes after ingestion of food.

Capecitabine doses were interrupted in patients with grade 3 or 4 diarrhea or other non-hematologic toxicities and were reduced for subsequent cycles. The paclitaxel dose on day 8 was reduced to 75% in patients with an absolute neutrophil count (ANC) of 0.5 to 0.99 × 10^9^/L and was omitted in patients with an ANC ≤ 0.5 × 10^9^/L; the dose was reduced to 50% for grade 3 non-hematologic toxicities and was omitted for grade 4 adverse events.

### 3. Dose Modifications for Adverse Events

Toxicity was evaluated before each treatment cycle according to the National Cancer Institute Common Toxicity Criteria (NCI CTC), version 3.0. To begin the next treatment cycle, each patient was required to have an absolute neutrophil count (ANC) ≥ 1.5 × 10^9^/L, a platelet count ≥ 100 × 10^9^/L and resolution of clinically significant non-hematological adverse events to grade 1 or 0.

Treatment was continued at the same dose, without interruption or dose reduction, in patients experiencing grade 1 or other toxicities considered unlikely to become serious or life threatening (e.g., alopecia). For all other treatment-related adverse events of grade 2 or higher, a dose modification scheme was implemented. Dose reduction was not required following the first appearance of any grade 2 toxicity, although treatment was delayed until the toxicity had resolved to grades 0-1. Treatment with both agents was interrupted and the dose of both agents was reduced by 20% in patients who experienced a second occurrence of any grade 2 toxicity or at the first occurrence of any grade 3 toxicity. If patients experienced a third occurrence of any grade 2 toxicity or a second occurrence of any grade 3 toxicity, treatment was interrupted/delayed until the toxicity resolved to grades 0-1 and the dose of both agents was further reduced by 20% of the previous dose. Treatment with both agents was discontinued if any grade 2 toxicity occurred for a fourth time or any grade 3 toxicity for a third time despite dose reduction. Treatment was discontinued if patients experienced a grade 4 non-hematologic toxicity. Paclitaxel was discontinued and capecitabine treatment was modified according to the scheme outlined above in patients with grade 3 peripheral neuropathy. The paclitaxel dose was reduced by 20% for patients who developed grade 4 neutropenia for more than 5 days or neutropenic fever.

### 4. Statistical Analysis

The primary objective of this study was to evaluate the response rate of paclitaxel plus capecitabine as second-line therapy in patients with metastatic or recurrent esophageal cancer. With the regard to the definition of therapy-line, the surgery and radiotherapy was not counted as therapy-line, only number of palliative chemotherapy was counted.

The Simon's two-stage optimal design was used for determining the total number of patients required for this phase II study. We set an overall response rate of 30% as the target activity level and 10% as the lowest overall response rate of interest. Our study was designed to have 90% statistical power with a 5% Type I error. With this design, 18 patients were enrolled at the first stage. If there were 2 or fewer responses out of the initial 18 patients, the study would conclude that the anticipated response rate is less than 10% and terminate. Otherwise, accrual continued to a total of 35 assessable patients. At the second stage, at least 7 objective responses among 35 patients were required for this regimen to be regarded as worthy of further investigation. Considering 10% follow up loss, 39 eligible patients would be enrolled. At the first stage, we observed a remarkably high response rate of 71% (5 partial responses among 7 patients). The protocol was therefore amended to estimate the response rate in patients with metastatic or recurrent esophageal cancer not only in the second-line setting, but also in the first-line setting. The response rate of the treatment was calculated as the ratio of the number of complete and partial responders to the total number of evaluable patients. A 95% confidence interval for the response rate was computed based on the binomial distribution function. Toxicity profile was assessed as the ratio of the number of occurrence to the total number of evaluable patients. The secondary objectives included time to progression and overall survival, which was estimated by the Kaplan-Meier method. Overall survival time was calculated from the first day of treatment to death or the last day of follow-up. Progression-free survival time was calculated from the first day of treatment to the date that disease progression or death from any cause was reported.

## Results

### 1. Patient Characteristics

Between February 2006 and February 2009, 32 patients with metastatic or recurrent esophageal cancer were enrolled at single institution. The baseline characteristics of the patients are summarized in Table 1. Twelve patients were chemotherapy-naïve. Twenty patients had received prior therapy including a platinum based regimen.

**Table 1:** Patient baseline characteristics

Characteristics		First-line		Second-line		All patients	(%)
					
		No	(%)	No	(%)		
Sex	Male	12	(100)	20	(100)	32	(100)
	Female	0	(0)	0	(0)	0	(0.0)
Age, median		58	(45-70)	66	(54-76)	60.5(45~76)	
ECOG PS	0	3	(25)	6	(30)	9	(28.1)
	1	9	(75)	13	(65)	22	(68.8)
	2	0	(0)	1	(5)	1	(3.1)
Histology	Squamous	12	(100)	20	(100)	32	(100)
Smoking history	Never	0	(0)	2	(10)	2	(6.3)
	Ever	12	(100)	18	(90)	30	(93.7)
Prior therapy	None	6	(50)	N/A		6	
	Surgery	6	(50)	4	(20)	10	
	Radiation	0	(0)	2	(10)	2	
	Chemotherapy	N/A		20	(100)	20	
No.of metastatic sites	1	3	(25.0)	5	(25)	8	(25.0)
	2	3	(25.0)	10	(50)	13	(40.6)
	3	1	(8.3)	4	(20)	5	(15.6)
Locoregional		5	(41.7)	1	(5)	6	(18.8)
Metastatic		7	(58.3)	19	(95)	26	(81.3)
Liver		3	(25.0)	13	(65)	16	(50.0)
Lung		7	(58.3)	19	(95)	26	(81.3)
Bone		2	(16.7)	5	(25)	7	(21.9)

Abbreviations: ECOG PS, Eastern Cooperative Oncology Group performance status; N/A, Not applicable.

The median age was 60.5 years (range; 45 to 76 years) and all patients were male. 97% of patients had an ECOG performance status of 0-1. Ten patients (31.3%) had undergone surgery. All patients were assessable for efficacy and safety analyses.

### 2. Treatment Exposure

The median number of cycles of PACE administered to patients was five for all patients. Reasons for treatment discontinuation were as follows; 27 patients discontinued therapy for disease progression, 2 patients for toxicity, 2 patients for non-compliance, and one patient continues treatment at the time of final analysis. The relative dose intensity of paclitaxel and capecitabine was 88.2% and 86.5%, respectively. The dose modifications were performed in only 11 (6.1%) of 180 cycles administered to all patients.

### 3. Efficacy

An overall response rate for the PACE regimen of 56.3% (95% CI, 39.0 to 73.4) was observed and the disease control rate was 75.0% (Table 2). Three patients achieved complete response (CR) and fifteen patients achieved partial response (PR). Among twelve patients receiving first-line treatment, two patients achieved CR and seven patients achieved PR, giving an overall response rate of 75% (95% CI, 50.5 to 99.5). Among twenty patients receiving second-line treatment, one patient achieved CR and eight patients achieved PR. The response rate of second-line PACE was 45% (95% CI, 23.2 to 66.8). Among twenty patients treated with second-line therapy, six patients (46.2%) who had a treatment free-interval of < 3 months between their last chemotherapy and PACE showed an objective response, which reflects the clinical efficacy in patients whose tumors progress more rapidly (Table 3). All twenty patients treated in the second-line received platinum-based chemotherapy in the first-line setting. With respect to prior chemotherapy received for patients treated in the second-line, two patients out of three treated with prior 5-FU/cisplatin and one patient out of six treated with docetaxel/cisplatin achieved partial responses to PACE. The clinical efficacy of PACE seems to be more slightly lower in patients previously treated with docetaxel, which suggest that paclitaxel may have cross resistance to docetaxel (Table 4). However, any definitive conclusions can not be made due to small sample size.

**Table 2:** Tumor response rate and survival according to therapy line

Efficacy endpoints	1^st ^line (n = 12)		2^nd ^line (n = 20)		Overall (n = 32)	
			
	**No**.	(%)	**No**.	(%)	**No**.	(%)
Complete response	2	(16.7)	1	(5.0)	3	(9.4)
Partial response	7	(58.3)	8	(40.0)	15	(46.9)
Stable disease	0	(0.0)	6	(30.0)	6	(18.7)
Progressive disease	3	(25.0)	5	(25.0)	8	(25.0)
Overall response rate	9	(75.0)	9	(45.0)	18	(56.3)*

*(95%Confidence Interval; 39.0-73.4)

**Table 3:** Tumor response by treatment free-interval in patients treated as second-line

Tumor Response	Treatment free-interval (N = 20)					
	
	< 3 months		3-6 months		> 6 months	
			
	**No**.	(%)	**No**.	(%)	**No**.	(%)
Complete response	1	(7.7)	0	(0)	0	(0)
Partial response	5	(38.5)	1	(33)	3	(75)
Stable disease	2	(15.4)	2	(67)	1	(25)
Progressive disease	5	(38.5)	0	(0)	0	(0)
Total	13	(100)	3	(100)	4	(100)

**Table 4:** Tumor response by prior regimen in patients treated as second-line

Tumor Response	Prior regimen (N = 20)			
	
	IP	FP	DP	NP
Complete response	0	0	0	1
Partial response	4	2	1	2
Stable disease	1	1	2	1
Progressive disease	2	0	3	0
**Total**	7	3	6	4

Abbreviations: IP, Irinotecan/cisplatin; FP, 5-fluorouracil/cisplatin; DP, docetaxel/cisplatin; NP, vinorelbine/cisplatin.

**Figure 1:**
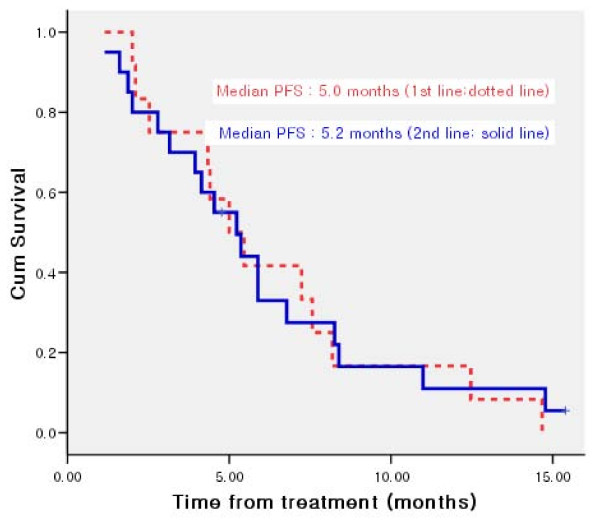
**Kaplan-Meier curve of progression free survival for patients treated as 1^st ^line and 2^nd ^line therapy**.

**Figure 2:**
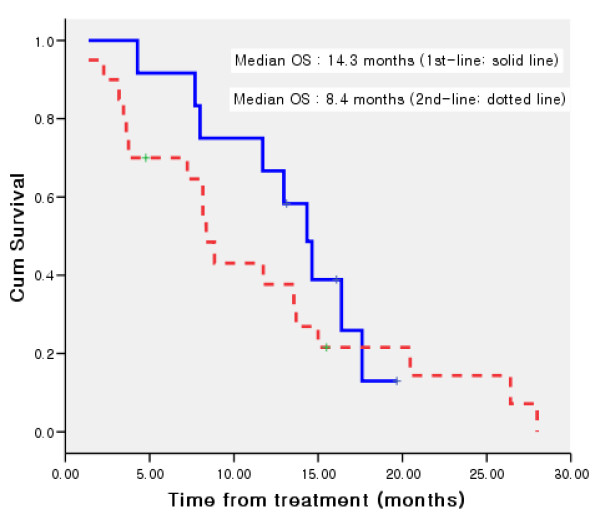
**Kaplan-Meier curve of overall survival for patients treated as 1^st ^line and 2^nd ^line therapy**.

Median progression-free survival was 5.2 months (95% CI, 4.0 to 6.5) (Figure 1) and median overall survival (OS) was 11.7 months (95% CI, 5.5 to 18.0) for all patients. The median OS was 14.3 months (95% CI, 10.6 to 18.0) for patients receiving therapy as 1^st ^line and 8.4 months (95% CI, 6.7 to 10.1) for those receiving as 2^nd^-line therapy (Figure 2).

### 4. Safety and Tolerability

All patients who received at least one cycle of study treatment were evaluated for toxicity (n = 32). The most common treatment related adverse events are listed in Table 5. Hematologic toxicity was common, with seventeen (53.1%) patients having grade 3-4 neutropenia and two (6.3%) patients febrile neutropenia. The most common grade 3 to 4 non-hematologic adverse events were stomatitis (n = 3), asthenia (n = 2), hand-foot syndrome (n = 1), and peripheral neuropathy (n = 1). No grade 4 non-hematologic toxicity was observed and there was no treatment-related death.

**Table 5:** Hematologic and non-hematologic toxicity (NCI-CTC version 3.0)

Toxicity		NCI-CTC Grade (n = 32)									
		0		1		2		3		4	
		**No**.	(%)	**No**.	(%)	**No**.	(%)	**No**.	(%)	**No**.	(%)
**Hematologic**											
	Leukocytopenia	4	(12.5)	9	(28.1)	11	(34.4)	8	(25.0)	0	(0.0)
	Neutropenia	5	(15.6)	3	(9.4)	7	(21.9)	10	(31.3)	7	(21.9)
	Anemia	1	(3.1)	12	(37.5)	17	(53.1)	2	(6.3)	0	(0.0)
	Thrombocytopenia	28	(87.5)	4	(12.5)	0	(0.0)	0	(0.0)	0	(0.0)
**Non-hematologic**											
Neurology	Neuropathy	18	(56.3)	8	(25.0)	5	(15.6)	1	(3.1)	0	(0.0)
GI	Stomatitis	13	(40.6)	12	(37.5)	4	(12.5)	3	(9.4)	0	(0.0)
	Anorexia	10	(31.3)	17	(53.1)	5	(15.6)	0	(0.0)	0	(0.0)
	Nausea/Vomiting	16	(50.0)	16	(50.0)	0	(0.0)	0	(0.0)	0	(0.0)
	Constipation	23	(71.9)	6	(18.8)	3	(9.4)	0	(0.0)	0	(0.0)
	Diarrhea	24	(75.0)	7	(21.9)	1	(3.1)	0	(0.0	0	(0.0)
Hepatic	AST↑	27	(84.4)	4	(12.5)	1	(3.1)	0	(0.0)	0	(0.0)
	ALT↑	29	(90.6)	3	(9.4)	0	(0.0)	0	(0.0)	0	(0.0)
	Bilirubin↑	25	(78.1)	6	(18.8)	1	(3.1)	0	(0.0)	0	(0.0)
Renal	Creatinine↑	23	(71.9)	8	(25.0)	1	(3.1)	0	(0.0)	0	(0.0)
Dermatology	Alopecia	5	(15.6)	16	(50.0)	11	(34.4)	0	(0.0)	0	(0.0)
	Rash	25	(78.1)	5	(15.6)	2	(6.3)	0	(0.0)	0	(0.0)
	Itching	27	(84.4)	5	(15.6)	0	(0.0)	0	(0.0)	0	(0.0)
	Hyperpigmentation	20	(62.5)	10	(31.3)	2	(6.3)	0	(0.0)	0	(0.0)
	Nail change	24	(75.0)	3	(9.4)	4	(12.5)	1	(3.1)	0	(0.0)
	Hand-foot syndrome	18	(56.3)	9	(28.1)	4	(12.5)	1	(3.1)	0	(0.0)
Others	Asthenia	8	(25.0)	16	(50.0)	6	(18.8)	2	(6.3)	0	(0.0)
	Myalgia	23	(71.9)	8	(25.0)	1	(3.1)	0	(0.0)	0	(0.0)
	Febrile neutropenia	30	(93.8)	0	(0.0)	0	(0.0)	2	(6.3)	0	(0.0)
	Pneumonitis	30	(93.8)	1	(3.1)	0	(0.0)	1	(3.1)	0	(0.0)

## Discussion

This prospective, phase II study provides important insights into the treatment of patients with esophageal squamous cell carcinoma. PACE showed promising efficacy, with an overall response rate of 75% as first-line, 45% as second-line and a median OS of 11.7 months. Treatment was well-tolerated and toxicity was manageable. Dysphagia is a common symptom in esophageal cancer, which may be cause of reduced number of eligible patients treated with capecitabine-based regimen. However, during study period, patients otherwise eligible who were excluded for dysphagia did not proceed the screening process and screening failure due to dysphagia was not observed. Oral administration of capecitabine is feasible and more convenient compared with infusional 5-FU in esophageal cancer.

Treatment of metastatic esophageal cancer still remains a serious challenge to medical oncologists. The most frequently used chemotherapy regimen is a combination of 5-fluorouracil and cisplatin, with response rates ranging from 20-45%. Recently, new agents such as taxanes, vinorelbine, irinotecan, capecitabine, and oxaliplatin have been investigated as single agent or in combination in esophageal cancer [6,14-17]. The results of other phase II trials using various agents in esophageal cancer are summarized in Table 6. Although direct comparison is difficult across several trials due to different clinicopathologic characteristics of patients, our study shows at least comparable efficacy or better outcomes than those of other studies.

**Table 6:** Results of phase II trials in esophageal cancer

Author	Regimen	Histology	Therapy line	Response rate (%)	TTP/PFS	OS
Lee [20]	XP (X:2,500 mg/m^2 ^D1-14 CDDP:60 mg/m^2^; D1)	SCC (n = 45)	1^st ^line (n = 45)	57.8%	4.7 mon	11.2 mon
Van Meerten [18]	XELOX (X:2,000 mg/m^2 ^D1-14 O:130 mg/m^2^; D1)	ADC (n = 45) SCC (n = 4) Undiff. (n = 2)	1^st ^line (n = 51)	39%	NR	8 mon
MB Polee [28]	Paclitaxel:180 mg/m^2^;D1 CDDP:60 mg/m^2^; D1	ADC (n = 31) SCC (n = 16) Undiff (n = 4)	1^st ^line (n = 51)	ADC (39%) SCC (44%)	NR	9 mon
Zhang [29]	Paclitaxel:175 mg/m^2 ^on D1 CDDP:75 mg/m^2 ^(D1)	SCC (n = 35)	1^st ^line (n = 35)	48.6%	7 mon	13 mon
S.Lorenzen [21]	DX (X:2,000 mg/m^2 ^D1-14 D:75 mg/m^2^; D1)	ADC (n = 7) SCC (n = 17)	1^st ^(n = 16) 2^nd ^(n = 8)	1^st ^(56%) 2^nd ^(25%) Overall = 46%	6.1 mon	1^st ^(15.8) 2^nd ^(6.2)
S.Lorenzen [26]	FP + Cetuximab vs FP	SCC (n = 62)	1^st ^line (n = 62)	FP+ Cetuximab (19%) FP (13%)	5.9 mon 3.6 mon	9.5 mon 5.5 mon
Current Study	Xeloda 1,800 mg/m^2 ^D1-14 Paclitaxel:80 mg/m^2 ^on D1, D8	SCC (n = 32)	1^st ^(n = 12) 2^nd ^(n = 20)	1^st ^(75%) 2^nd ^(45%) Overall = 53.6%	5.23 mon 4.54 mon	14.3 mon 8.4 mon

Abbreviations: XP, capecitabine/cisplatin; XELOX, capecitabine/oxaliplatin; DX, docetaxel/capecitabine; FP, 5-fluorouracil/cisplatin; SCC, squamous cell carcinoma; ADC, adenocarcinoma; Undiff, undifferentiated carcinoma; TTP, time to progression; PFS, progression-free survival; OS, overall survival; NR, not reported.

Several capecitabine-based regimens were evaluated in the first-line settings. Van Meerten et al [18] reported that capecitabine and oxaliplatin was an active regimen with a response rate of 39% and median OS of 8 months in the first-line. In that study, 45 of 51 patients (88%) had a histologic diagnosis of adenocarcinoma, whereas all patients in our study had squamous cell histology. Another study demonstrated the efficacy of oxaliplatin and capecitabine in patients with esophageal squamous cell carcinoma (ESCC), with a response rate of 43.8% and a median OS of 10 months [19]. Although it is known that there are biological differences between esophageal adenocarcinoma and squamous cell cancer, clinical efficacy of capecitabine and oxaliplatin is not different between adenocarcinoma and squamous cell carcinoma of the esophagus. Lee et al [20] also reported the results of cisplatin and capecitabine (XP) in ESCC. The response rate was 57.8% and median OS was 11.2 months. The authors concluded that the XP regimen was a promising combination chemotherapy in metastatic esophageal squamous cell carcinoma with a tolerable toxicity profile. Another docetaxel/capecitabine trial reported a response rate of 46% and median survival of 15.8 months [21]. However the incidence of hand-foot syndrome (HFS) and diarrhea was higher than that of our study, which can be explained by the lower dose of capecitabine (900 mg/m^2 ^twice daily) in our study. Another explanation for lower rate of HFS in our study is that the risk factors for HFS have been reported to include use of docetaxel chemotherapy and ethnic differences may also contribute to the occurrence of HFS [22]. Randomized phase II study of weekly docetaxel (30 mg/m^2^) on days 1 and 8 and capecitabine (1600 mg/m^2 ^per day) on days 1-14 showed a response rate of 26%, median PFS of 4.6 months, and OS of 10.1 months in patients with esophagogastric cancer. However, in this study, only 36% of patients were esophageal cancers and 16% of patients had squamous cell carcinoma. The confirmed response rate was 26%, which was lower than that of our study, in which only unconfirmed response rate was reported. The more than grade 3 HFS was observed in 5% of patients treated with docetaxel and capecitabine [23].

Paclitaxel-based regimens were evaluated in patients with esophageal cancer. The result of a phase II trial of paclitaxel and nedaplatin as first line chemotherapy for advanced esophageal cancer was reported, with a response rate of 41.7%, median time to progression of 6.1 months and median overall survival of 11.5 months [24]. In this study, 46 of 48 patients had squamous cell histology and 2 patients had adenocarcinomas. Another study using paclitaxel and nedaplatin as first line therapy showed that the overall response rate was 43.6%, median progression-free survival and median overall survival was 6.1 and 10.3 months, respectively [25]. Among 39 enrolled patients, 36 (92.3%) had squamous cell carcinoma. Similar to metastatic disease, in unresectable locally advanced esophageal cancer (squamous/adenocarcinoma; 36/14), definitive concurrent chemoradiation with weekly paclitaxel and carboplatin also showed promising efficacy with OS of 17 months and median time to local progression of 14 months, which demonstrated high clinical activity of paclitaxel-based regimen.

However, median overall survival rarely exceeds 12 months despite the improvement of chemotherapy regimens and supportive care in metastatic esophageal squamous cell carcinoma. There are several strategies to improve the overall survival of metastatic solid tumors, such as adding another cytotoxic agent to doublet regimen, thus using triplet therapies and adding molecular targeted agents to cytotoxic chemotherapy. As shown in many solid tumors, several clinical trials with triplet chemotherapy (taxane, platinum and 5-FU) demonstrated no superiority to historical doublet chemotherapy regimens in metastatic esophageal cancer,[2] although no direct comparison is possible across the trials. Regarding molecular targeted agents, a randomized phase II study comparing the epidermal growth factor receptor (EGFR) inhibitor, cetuximab in combination with 5-FU and cisplatin (FC), with FC alone was conducted to evaluate the efficacy and safety in first-line metastatic squamous cell carcinoma of the esophagus [26]. The median PFS (5.9 versus 3.6 months) and the median OS (9.5 versus 5.5 months) both favored the cetuximab plus FC combination, but the median OS of 9.5 months was disappointing despite the addition of cetuximab to chemotherapy, considering the median OS ranging between 8 and 15 months reported in several phase II trials with chemotherapy alone [6,20,21,27].

Maintaining quality of life (QOL) and symptom relief are important factors in the management of patients with metastatic solid tumors. In this study, we did not evaluate QOL, including dysphagia, during study period, but the toxicities were generally well-tolerated for most patients. The most common reason for treatment discontinuation was disease progression, whereas only two patients stopped therapy due to toxicity.

Compared with other studies, the observed response rate, although not confirmed at least 4 weeks after initial response evaluation, was higher than our expectations at the time of study design despite administering treatment as second-line therapy, so the patients who were not previously treated with any chemotherapy were also included in this study. However, although change of patients population was another weak point of our study, at the first stage, we observed a remarkably high response rate of 71% (5 partial responses among 7 patients) and considering that the incidence of esophageal cancer is relatively low and patients recruitment was slow, we enrolled the patients in the first-line setting. With regard to protocol amendment, we did not consider statistical correction.

The median OS was 14.3 months for patients receiving therapy as 1^st^-line and 8.4 months for those receiving as 2^nd^-line therapy. Although the small sample size of patients treated as 1^st^-line makes it difficult to draw definitive conclusions, this regimen deserves further evaluation as front-line treatment for esophageal cancer considering the high clinical activity observed in our study.

## Conclusions

Weekly paclitaxel and capecitabine treatment as first- and second-line therapy is highly active and well-tolerated in patients with advanced esophageal squamous cell carcinoma. Non-platinum based doublet chemotherapy showed significant clinical benefit in our study, and further studies using non-platinum-based chemotherapy are warranted for the treatment of esophageal cancer. Ultimately, randomized clinical trials are needed to determine the efficacy and safety of paclitaxel and capecitabine for esophageal cancer patients.

## Conflict of interest disclosures

The authors declare that they have no competing interests.

## Authors' contributions

HTK: Study design, interpretation of data and preparation of the article for publication.

TY: Collection of data, interpretation of data and writing the manuscript. HLC: Collection of data. HYK: Implementation of the radiologic response. BHN: Performing the statistical analysis. JYH, JSL: Collection of data and interpretation of data.

All authors read and approved the final manuscript.

## Pre-publication history

The pre-publication history for this paper can be accessed here:

http://www.biomedcentral.com/1471-2407/11/385/prepub
